# The Influence of Suspension Containing Nanodiamonds on the Morphology of the Tooth Tissue Surface in Atomic Force Microscope Observations

**DOI:** 10.1155/2018/9856851

**Published:** 2018-11-05

**Authors:** Helena Gronwald, Katarzyna Mitura, Lukas Volesky, Pavel Kejzlar, Michał Szczypiński, Elżbieta Kubala, Paulina Strzelecka, Marta Grzegocka, Piotr Baszuk, Piotr Skomro, Danuta Lietz-Kijak, Stanisław Mitura, Petr Louda, Totka Bakalova

**Affiliations:** ^1^Independent Unit of Propaedeutics and Dental Physicodiagnostics, Pomeranian Medical University Szczecin, Al. Powstańców Wielkopolskich 72, 70-111 Szczecin, Poland; ^2^Faculty of Technology and Education, Koszalin University of Technology, Ul. Śniadeckich 2, 75-453 Koszalin, Poland; ^3^Institute for Nanomaterials, Advanced Technologies and Innovation, Technical University of Liberec, Studentská 1402/2, 46117 Liberec 1, Czech Republic; ^4^Department of Material Science, Technical University of Liberec, Studentská 1402/2, 46117 Liberec 1, Czech Republic; ^5^Doctorate Studies, Pomeranian Medical University, Szczecin, Ul. Rybacka 1, 70-204 Szczecin, Poland; ^6^Department of Genetics and Pathology, Pomeranian Medical University, Ul. Unii Lubelskiej 1, 71-252 Szczecin, Poland; ^7^Department of Electroradiology, State School of Applied Sciences, Ul. Nowy Świat 4, 62-800 Kalisz, Poland

## Abstract

Reduced friction and wear of materials after the use of the carbon nanomaterials including nanodiamonds (NDs) have been confirmed by several studies in material engineering. Mechanical cleaning of the tooth surface by brush bristles should leave as little tissue roughened as possible. Higher surface roughness increases the tissue's wear and encourages the redeposition of the bacteria and the colouring agents present in the diet. Therefore, we evaluated the tooth tissues' surface's morphological changes after brushing them with the NDs suspension. Ten human teeth were brushed with the NDs aqueous suspension. The surfaces were observed using an Atomic Force Microscope (AFM). We found that the nature of the tissue surface became milder and smoother. A number of selected profilometric parameters were compared before and after brushing. We observed that brushing with the suspension of NDs resulted in a significant reduction in the enamel and dentine's surface roughness both in the range of the average parameters (Ra; p-0,0019) and in the detailed parameters (Rsk; p-0,048 and Rku; p-0,036). We concluded that the NDs used in the oral hygiene applications have a potentially protective effect on the enamel and the dentine's surfaces.

## 1. Introduction

With the development of material engineering, the amount of mechanical and chemically active agents on the hard tissue of the tooth increases. At the same time, questions arise about the effect of these substances on the surface of tissues [1]. The dental structure degradation, as well as changes occurring on the surface of the tooth tissues, depends on genetic conditions; age, diet, hygiene, and are a major problem in dentistry but also in related biomaterial sciences. Intentional impact of biomaterials on the surface during processing, preventive treatment, or nutrition is an important step in preserving or restoring their properties [2]. The current technological progress in materials science has resulted in the creation of a new branch of surfometry that allows for the morphological, quantitative metrology of the tissue surface [3, 4]. Surface morphology is extremely important when analyzing physical phenomena such as adhesion, wettability, and wear. It also facilitates understanding of biological phenomena such as physiological and pathological tissue wear, dental hypersensitivity resulting from tissue loss [5]. Noncarious cervical lesions are seen daily by clinicians in dental practice [6]. Incorrect cervical changes are often caused by mechanical interactions between the toothbrush and the tooth and toothpaste and tooth, leading to loss of substance. The increase in the loss of tissue which occurs as a result of personal hygiene is also affected by the increase in the force of friction. This depends on the patient as well as on the addition of abrasives and detergents in toothpaste. Other cofactors are chemical erosion and the bite overload [7, 8]. In modern dentistry, the use of minimally invasive methods of hygiene and dental procedures conducive to retaining its own tooth tissues is paramount. Because of the constant and active presence of dental plaque dental brushing cannot be dispensed with. However, it is necessary to elaborate the method reducing friction and decreasing loss of enamel and dentin. An example of a substance added to a toothpaste to reduce its abrasivity is polysaccharide chitosan [9].

Mechanical cleaning of the tooth surface by the brush bristles should leave as little tissue roughened as possible to impede the redeposition of the bacteria and the colouring agents present in the diet because it leads to caries and discolouration. In 2015 Dickson et al. showed that brushing with toothpaste resulted in significantly greater cervical tooth loss than brushing with water, which was significantly greater than no treatment [10]. Increasing the roughness of the enamel surface after brushing with distilled water was caused by exposing the sharp crystalline peaks to hydroxyapatite, which leads to the surface wearing away faster. In addition to the development of surface metrology over the last twenty years, there has been a significant increase in the number of studies on carbon nanomaterials and their impact on friction reduction and wear prevention [11]. The use of carbon nanomaterials in coatings was investigated. They were also applied in lubricants as a component to reduce friction and wear [12]. In comparison with traditional, environmentally hazardous additives, the use of nanodiamonds lubrication has an advantage because of their nontoxicity and chemical stability. Several lubricating mechanisms of the nanodiamonds function have been suggested including the sliding with a ball-bearing effect, the formation of a tribofilm, and the polishing effect [13]. It should be noted that, in parallel with the study of mechanical properties and chemical durability, the biocompatibility of diamond powder particles (DPP) was also tested [14]. Development of observation methods such as SEM with EDS, AFM, and XRD detection opens new perspectives for understanding tissue mineralization. In addition, the relationship between medicine and physics offers an opportunity to conduct interdisciplinary research. Reduced friction and wear of materials after the use of NDs have been confirmed by several studies in material engineering; however, there are no data on tooth tissue surface morphology after NDs application (Table 1). We, therefore, decided to evaluate the influence of brushing with NDs suspension on tooth tissues surface morphology.

## 2. Methodology

**Figure 1:**
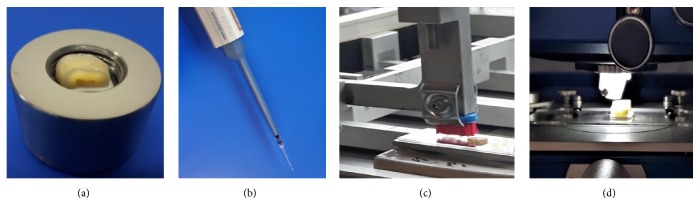
The sample of the tooth prepared for grinding and polishing (a), application of the NDs suspension (b) the tooth brushing simulation (c), and the AFM observations (d).

Twenty human teeth, healthy permanent third molars, extracted from patients aged 20-40 years for orthodontic reasons were collected according to the protocol KB-0012/88/17 approved by the Bioethics Committee of Pomeranian Medical University in Szczecin, Poland. Ten of the collected teeth were selected for testing. The inclusion criterion was teeth with healthy enamel and dentine at the same level of mineralization. The exclusion criteria were molars with restorations, dental caries, the presence of enamel hypoplasia, or demineralization. The material was analyzed by the fluorescence method DIAGNODent, (KaVodental, Biberach, Germany) and the ten selected teeth (values less than 9) qualified for the further study. These molars were stored for a week in 0.9% NaCl solution at 4°C before the study commenced. The procedure of enamel and dentin sample preparations was carried out in accordance with ISO 11609: 2010 [15]. A slow-speed water-cooled diamond saw IzoMet 1000 (Buehler Ltd., Lake Bluff, IL, USA) was used to remove the roots of the teeth by cutting centrally, subsequently cleaving them mesial-distal along the long axis. The obtained half teeth were ground flat with water-cooled discs 1200, 2400 and 4000 grit, waterproof silicon carbide paper (Struers, Erkrath, Germany), and semiautomatic grinding and polishing Teragmin (Struers GmbH, Willich, Germany) to get flat, parallel samples (Figure 1(a)) necessary for AFM observation. To enable the in vitro studies to be carried out as closely as possible to the clinical conditions, samples were not embedded in epoxy resin so as not to damage (denature) the proteins contained therein but were stabilized on glass plates using a power bond tape (Tesa Tape Ltd., Poznań, Poland) and an automatic micropipette was used to dispense the measured amount of NDs.

**Figure 2:**
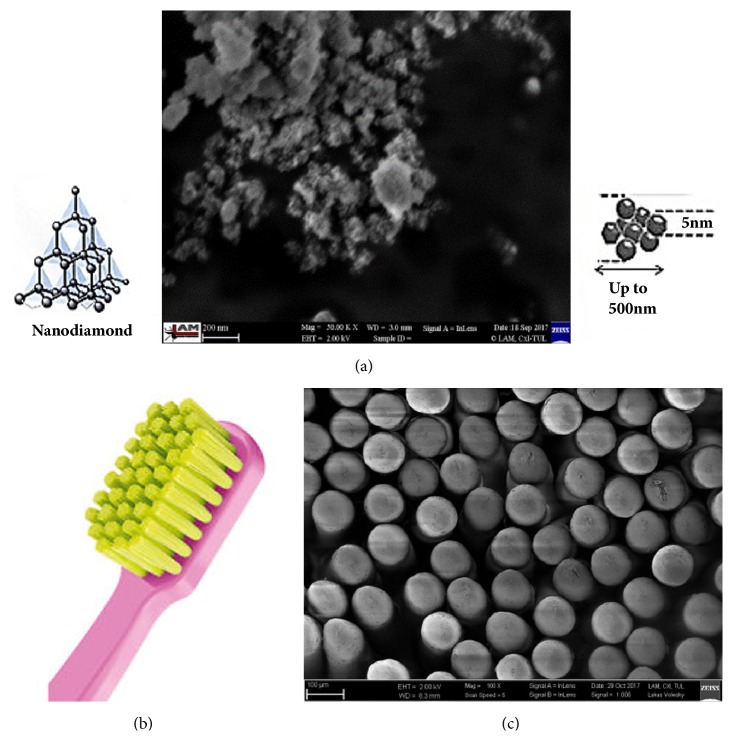
NDs powder-SEM image (a), head of toothbrush (b), and polymers of toothbrush-SEM image (c).

Five of the ten teeth were assigned to preliminary studies to establish reproducible polishing of samples, the number of front-back brush cycles, the minimum load necessary to ensure continuous brush contact with the surface of the sample, and the volume of the NDs suspension. The remaining five teeth were assigned for the main study. The samples with marked areas for measurements of enamel and dentin were stored in deionized water. Half of the polished tooth surface was covered with PVC adhesive tape (Tesa Tape Ltd., Poznań, Polska) to form a reference plane (a control group) unbrushed with NDs suspension [16]. 1% aqueous suspension of NDs was prepared just before application on the sample's surface (2-5 nm detonation NDs powder, New Technologies, Chelyabinsk, Russia) (Figure 2(a)) [17]. The shape and size of NDs were assessed using an FE SEM ULTRA plus (field emission scanning electron microscope, Carl Zeiss NTS GmbH, Germany) (Figure 2(a)).

Elkometer 1720 washability tester (Figure 1(c)) was used for dental brushing simulation carrying out 200 brush cycles with a load of 300g. 20*μ*l NDs suspension was also used for each sample, as well as a new ultra-soft toothbrush Curaprox 5460 with the 100*μ*m diameter of Curen® filaments (Curaden Ltd., Wrocław, Poland) (Figures 2(b) and 2(c)).

After brushing, the samples were washed with distilled water in an ultrasonic cleaner, and the adhesive tape was removed. The brushed and the reference surfaces of each sample were observed in AFM (FRT, Bergisch Gladbach, Germany) (Figure 1(c)).

**Figure 3:**
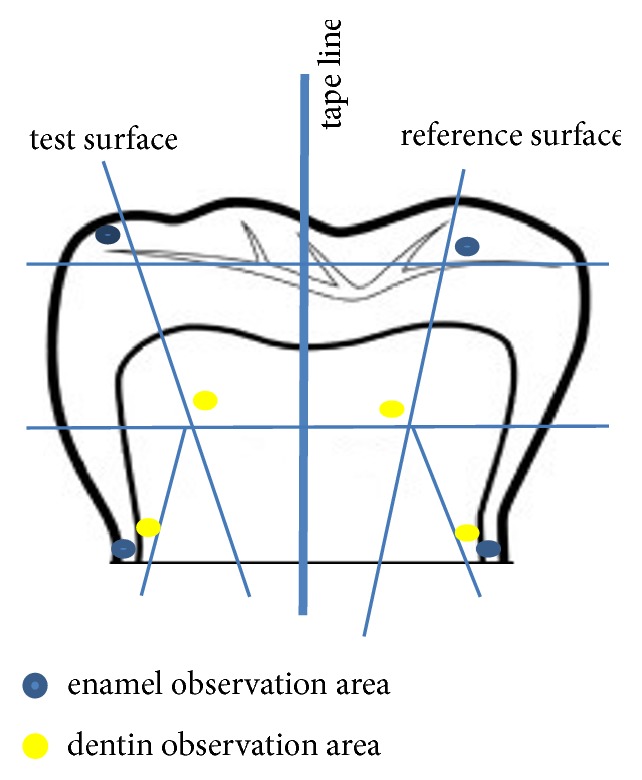
Twenty enamel and dentine observation areas on the study group and twenty corresponding areas on the reference surfaces.

JPK Data Processing for JPK Instruments AG software and Gwyddion 2.48 were used for data analysis. Twenty enamel and dentine areas of the brushed surfaces, as well as twenty enamel and dentine areas of the reference surfaces, were indicated for AFM measurements (Figure 3).

The material was divided into five groups (1-5) and selected—Ra, Rsk, Rku—profilometric parameters were calculated for the study and reference groups, distinguishing between dentine and enamel. The calculations were performed using the R statistical environment (R version 3.3.3 (2017-03-06) – “Another canoe” Copyright (C) 2017 The R Foundation for Statistical Computing Platform: x86_64-w64-mingw32 / x64 (64-bit). The following tests were used to compare the differences between the examined groups: Mann-Whitney, where the distribution of data for at least one comparison group was different from normal (normal test tally by Anderson Darling test was used but for an observation of less than 7, the Shapiro-Wilk test was used), Student T-test, if the data distributions of both groups were normal. The data was considered as paired because it is related to the same material with and without the use of NDs. For this reason, Wilcoxon's nonparametric test was used; p-value <0.05 was considered as statistically significant.

## 3. Results and Discussion

**Figure 4:**
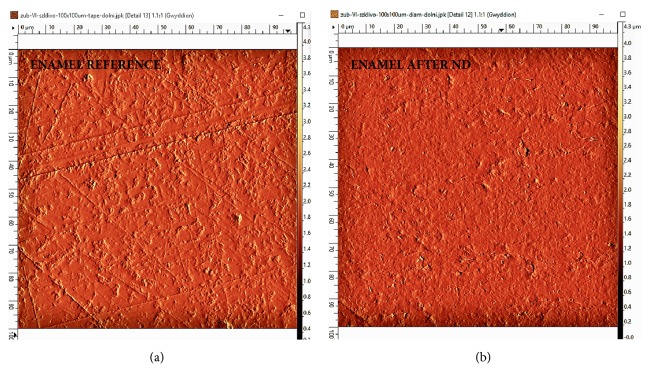
The AFM observations of enamel in the control group (a) and study group (b).

**Figure 5:**
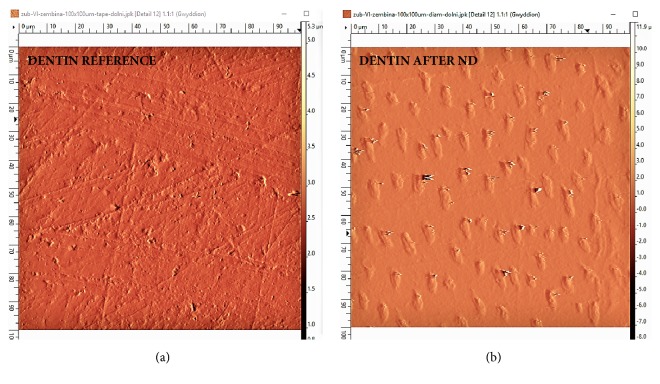
The AFM observations of dentin in the control group (a) and study group (b).

The AFM enamel and dentin observations were made for the control (a) and study (b) groups (Figures 4 and 5).

The enamel surface after brushing with NDs is smooth and the traces of the previous preparations are almost completely invisible (Figures 4(a) and 4(b)).

After brushing with NDs (Figure 5(b)), the dentin surface observed between the dentinal tubule orifices is much smoother. The orifices of the dentinal tubules are more visible than on reference dentin due to the removal of the smear layer (Figures 5(a) and 5(b)). The traces of the previous preparation disappeared after brushing (Figure 5(b)).

Profilometric measurements were made based on the AFM images. Selected profilometric parameters were calculated: Ra (average roughness), the arithmetic mean of the height of peaks and depth of the valleys from a mean line. This parameter describes the overall surface roughness [18]; Rku (kurtosis), it characterizes the flatness of the surface distribution [19]; Rsk (skewness), it characterizes the asymmetry of the surface distribution.

**Figure 6:**
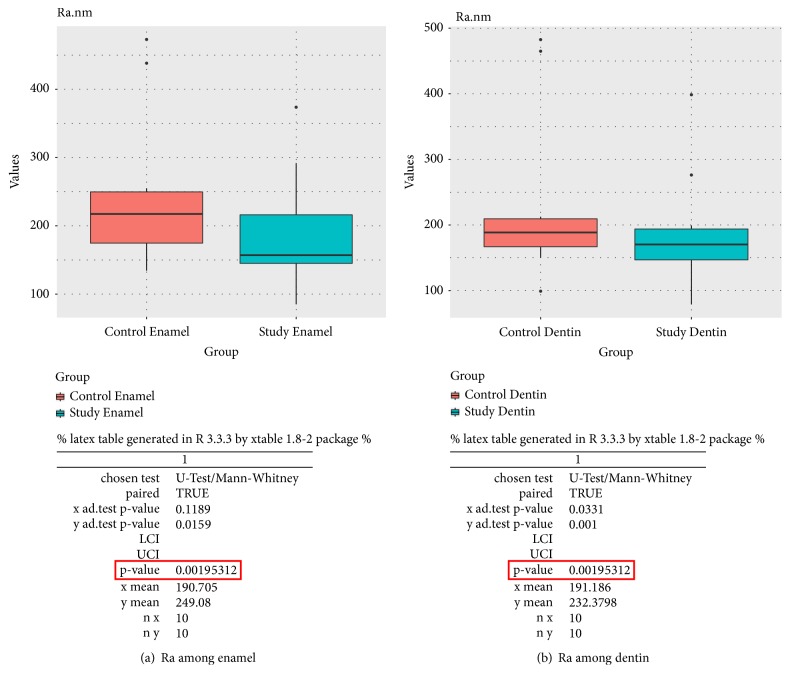
The box plots above display the comparison of Ra parameters for enamel (a) and dentin (b) between the control and the study group, as well as the detailed calculations.

**Figure 7:**
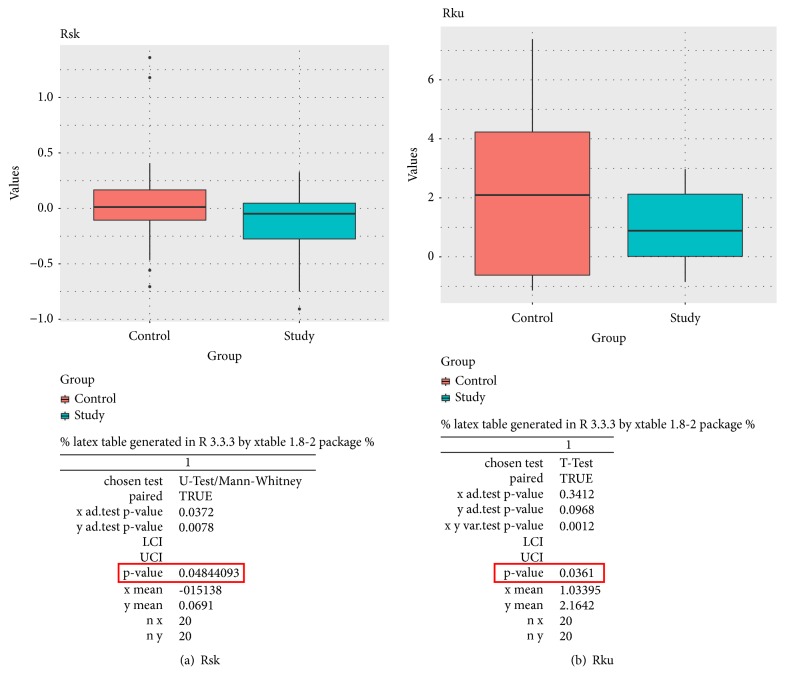
The above box plots display the comparison of the detailed parameters: Rsk (a) and Rku (b) between the control and the study group, as well as the detailed calculations.

The box plots below display the comparison of Ra parameters for enamel (a) and dentin (b) between the control and the study group, as well as the detailed calculations (Figure 6) and the comparison of Rsk and Rku parameters between control and study group the detailed calculations respectively (Figure 7). We observed a significant reduction in surface roughness both in the range of the average (Ra) and the detailed (Rsk and Rku) parameters for the enamel and the dentine after brushing with a suspension of NDs (Figures 6 and 7). Meanwhile, the literature data indicate that the surface roughness increases and there is increased loss of tooth structure after brushing either with toothpaste or with distilled water [9, 10]. The reduction in roughness observed in our study may be due to the presence of NDs between the brush polymer and the tooth tissues.

This may affect the occurrence of rolling-sliding effects alongside the polishing. This results in reduced surface roughness observed in the range of the average and the detailed parameters. The mean roughness of a surface influences its tribological behaviour. As the mean roughness (Ra) increases, the friction and the wear increase as well, as observed in Kumar's research [20]. This behaviour is observed equally when the teeth are brushed either with toothpaste or with distilled water [21]. Particularly prone to the phenomenon of tissue loss caused by brushing are patients with good dental hygiene (with a small amount of dental plaque covering the surface of the teeth) who repeatedly put their teeth under a heavy load, e.g., as a result of bruxism. The parameter Ra reflects the surface topography, but the additional information about the geometric structure of the surface is provided by a combination of Rsk skewness and Rku kurtosis. The combination of these parameters is useful to describe the shape of the topographical height distribution and its effect on the friction behaviour [22]. Skewness is the measure of the asymmetry of surface deviations with reference to a mean plane. Kurtosis is the measure of the peakedness or sharpness of the height distribution topography [23]. The parameter Rsk skewness describes the surface topography and mutual relations of valleys and heights. In our study, the negative Rsk value for samples from the research group (after NDS) indicates that the valleys dominate in the surface topography, which is associated with a greater ability to hold liquid on the surface. The predisposition to keep the liquid on the surface of the tissues is significant, for example, due to the presence and protective action of saliva in the oral cavity. A positive Rsk value for control group samples indicates that surface topography without contact with NDs is dominated by sharp peaks. This causes less predisposition to retain liquid on the surface and greater susceptibility to wear.

The observed decrease in Rsk and Rku coefficients indicates that the surface roughness of the tissues after brushing with the NDs suspension was softened. It also indicates that the nature of the tissues' points dis-plays more recesses than sharp peaks. Interestingly, Elomaa et al. showed that the friction and the wear properties of the agglomerated NDs were observed to be statistically better when compared to those of the evenly distributed NDs [24]. Our results obtained with the use of the nonstabilized water NDs suspension that contains the NDs agglomerate confirm Elomaa's observations also with respect to the surface of the tooth tissue.

Toothbrushing frequency and force, as well as toothbrush hardness, were shown to act as cofactors in the multifactorial aetiology of noncervical carious lesions. In vitro studies showed that toothbrushing abrasion is primarily related to the abrasivity of the toothpaste, while the toothbrush acts as a carrier, only modifying the effects of the toothpaste [25]. Therefore, it is justified to search for substances that, if used in oral hygiene products, would reduce the roughness and, consequently, the wear of hard tooth tissues.

The AFM observation method we used is a valuable method of assessing the effects of different substances or procedures used in the oral environment. This method allows us to determine the qualitative structural changes in the enamel and the dentine samples [26, 27].

The AMF has an important advantage: it allows for a noninvasive mapping of the high-resolution surface properties. Recently, more attention has been focused on the application of AFM in dental research to explore biomaterial surfaces [28]. AFM is useful because it has a higher resolution than SEM and three-dimensional images can be obtained [29]. Our observations were performed without any damage by contact profiler or any changes resulting from the use of a deep vacuum in SEM [4].

The difference in surface morphology could be observed due to the use of profilometric measurements separately for the enamel and the dentin (this is particularly important for the average parameter Ra). This, consecutively, was made possible by the heterogeneity of the tissues. Biominerals may be observed under the “solid-state” methodologies. In this domain, enamel is the best inorganic candidate to be characterized by the materials science techniques. Enamel is perfect for calibrating physicochemical investigations. Indeed, enamel is the most mineralized biological tissue (97% mineral) and is based on a noncollagenous matrix scaffold, in contrast to bone or dentin [30]. The observed reduction of the tissue's roughness after the application of NDs was made possible by the adoption of the same tooth's second half as a control group. Before the NDs were applied to one of the halves, both of them had the same roughness parameters because they were subjected to the same preparatory procedure. This way of selecting the control group limited the effect of individual variability on the measurement results. This methodology of observations had been applied by other authors [9].

There are limitations to our study. The number of cases is relatively small; however, we were able to obtain statistical significance. In this study, the influence of the saliva was not taken into account, which is worth considering when continuing experiments. The different functions of the saliva are related to maintaining oral health and protection of hard dental tissues [31]. This type of in vitro study using NDs helps in the assessment of abrasion caused by daily brushing of teeth before planning costly and time-consuming clinical trials.

## 4. Conclusions

NDs used in the oral hygiene measures have a potentially protective effect on the enamel and the dentin surfaces.

## Figures and Tables

**Table 1:** The number of articles in 2012-2017r. associated with “nanodiamond, biotribology, dental surface, and metrology” searched in abstracts, titles, and keywords.

No.	Keyword	Number of papers in PubMed	Science Direct
	*1 keyword*		
1	Metrology	4,176	26,495
2	Dental surface	29,888	6,763
3	Nanodiamond	597	3,040
4	Bio- tribology	32	1,514
	*2 keywords*		
1	Dental Surface + Metrology	12	199
2	Dental Surface + Bio- tribology	1	139
3	Dental Surface + Nanodiamond	8	70
4	Nanodiamond + Metrology	2	28
5	Nanodiamond + Bio- tribology	0	11
	*3 keywords*		
1	Dental Surface + Nanodiamond + Bio- tribology	0	0
2	Dental Surface + Nanodiamond + Metrology	0	0

## Data Availability

(1) The profilometric row data used to support the findings of this study have been deposited in the LHN server repository [lhn-server.cxi.tul.cz] of Technical University of Liberec, Czech Republic. (2) The elaborated profilometric data calculated on the basis of row data used to support the findings of this study are included in the article.

## Nomenclature

NDs: Nanodiamonds
DPP: Diamond powder particles
Ra (nm): Arithmetic mean height, mean surface roughness
Rku: Kurtosis of the surface height distribution. It characterizes the flatness of the surface distribution and corresponds to the fourth moment of the density function
Rsk: Skewness of the surface height distribution. It characterizes the asymmetry of the surface distribution and corresponds to the third moment of the density function
AFM: Atomic Force Microscope
SEM with EDS: Scanning Electron Microscope with Energy Dispersive Spectroscopy
XRD: X-ray diffraction.
